# Seasonality and Biting Behavior of *Mansonia* (Diptera, Culicidae) in Rural Settlements Near Porto Velho, State of Rondônia, Brazil

**DOI:** 10.1093/jme/tjac016

**Published:** 2022-02-21

**Authors:** Allan Kardec R Galardo, Andréa V Hijjar, Liliane Leite O Falcão, Dario P Carvalho, Kaio Augusto N Ribeiro, Guilherme A Silveira, Noel Fernandes S Neto, José F Saraiva

**Affiliations:** 1 Instituto de Pesquisas Científicas e Tecnológicas do Estado do Amapá – IEPA, Laboratório de Entomologia Médica, Núcleo de Biodiversidade, Rodovia Juscelino Kubitschek, Km-10, Fazendinha, CEP 68903-419, Macapá, AP, Brazil; 2 SAPO - Saneamento Ambiental Projetos e Operações, Rua Alexandre Guimarães, n. 4600, Agenor de Carvalho, CEP 76820-208, Porto Velho, RO, Brazil; 3 Santo Antônio Energia – SAE, Rodovia BR365, Km 9 (Zona rural), Porto Velho, RO, Brazil; 4 FUNDUNESP – Fundação para o Desenvolvimento da UNESP, Rua Líbero Badaró, nº377, 23º Andar, Conjunto 2310, Centro, CEP 01009-906, São Paulo, SP, Brazil

**Keywords:** exophilic, mosquitoes, visiting behavior

## Abstract

*Mansonia* (Diptera: Culicidae) are known to cause discomfort to the local populations of Amazon. Considering the fact that the effective control of these mosquitoes can only be obtained by understanding their ecology and behavior, entomological monitoring becomes essential. In view of this, mosquitoes of the genus *Mansonia* were collected by human landing catches (HLC) from 2015 to 2019, in four locations of Porto Velho, Rondônia, Brazil. The collections were performed inside and outside the homes, once in every four months, uninterrupted for 24 hr. Human bite indices/hour was used to analyze the hourly activity of the species for different seasons and environment (indoor and outdoor). Moreover, nonparametric Mann–Whitney tests were conducted to indicate if there were differences between exophagic and endophagic behavior. The seasonality of *Mansonia* species was also analyzed. Overall, 96,766 specimens were collected over five years of sampling. *Mansonia titillans* (Walker) was found to be the most abundant species (76.9%). The highest percentage of mosquitoes was collected in February (48.4%), followed by October (39.6%) and June (12.0%). The biting activity of the two most abundant species showed peak host seeking activity/behavior during twilight and night, more perceptible in the outdoor environment (peridomiciliary). In general, seasonality showed a tendency towards a reduction in the abundance of *Mansonia* in the years after 2015. Our results will be essential in the formulation of effective control methodology for *Mansonia* in the studied area.

*Mansonia* Blanchard (1901) are large to medium-sized mosquitoes, with a truncated abdomen and a body covered by broad intercalated dark brown and yellowish scales, giving the wings an asymmetrical appearance (Harbach 2019). The genus is classified into two subgenera, *Mansonia* and *Mansonioides*. The subgenus *Mansonia* only occurs in the New World, while the subgenus *Mansonioides* occurs in the Old World (Ronderos and Bachmann 1963, Darsie and Ward 2005, Harbach 2019). Most species are adapted to tropical climates, which explains their diversity in the neotropical region, where 15 species have been recorded (Harbach 2019, WRBU 2020).

The most notable characteristic of *Mansonia* is the adaptation of the spiracular apparatus (siphon and trumpet) in larvae and pupae that allows their attachment to the submerged parts of aquatic plants to obtain oxygen from the aerenchyma sacs (Laurence 1960, Belkin et al. 1970). Some species of *Mansonia* are more general in terms of their preference to the variety of aquatic plants, whereas others are species-specific (aquatic plant) (Chandra et al. 2006). Macrophytes of the genus *Pistia* (water lettuce) are the most common plants associated with *Mansonia*. Immature stages usually occur in permanent aquatic habitats that are densely covered by macrophytes (Forattini 2002, Rattanarithikul et al. 2006). In general, the control of mosquitoes requires choice of methodologies based on entomological monitoring data, obtained through field monitoring of the species, that allows more effective control of important species (Becker et al. 2010).

*Mansonia* are aggressive biters, causing serious discomfort to their hosts (Tadei et al. 1991, Harbach 2019). Hematophagic activity is predominantly nocturnal, with peaks at morning and dusk (Harbach 2019), feeding on both humans and domestic or wild mammals (Luz and Lourenço-de-Oliveira 1996, Kengne et al. 2003). Females are strongly attracted by artificial light, but predominately bites in outdoor (exophilic) environment, with only exploratory indoor activities (or endophilic), characterized as visiting behavior (Tadei et al. 1991, Navarro-Silva et al. 2004).

The visiting behavior (endophilic) in *Mansonia* is still controversial. It was noticed from continued observations in the Southeast region of Brazil that the anthropic changes in the environment, favor the populations of these mosquitoes (Forattini and Massad 1998). Thus, after anthropic modifications such as the installation of an artificial irrigation system or the clearing of the land for agricultural purposes, a stimulus of increased indoor biting has been observed. Hence, it strengthens the hypothesis of the presence of a high adaptive capacity to supervening conditions combined with a considerable power of dispersal. Moreover, the communities closer to the breeding sites showed more abundance of mosquitoes and higher rate of mosquito bites (Rueda 2007).

Although the entire Amazon region is favorable to the proliferation of these mosquitoes, entomological monitoring studies to control *Mansonia* have not been carried out yet. The present study aimed to identify the species of the genus *Mansonia* occurring in four locations of Porto Velho, determine the dominant species in the affected settlements, monitor populations to verify seasonal patterns, and study the biting behavior inside and outside the households. The selection of the studied areas was based on the abrupt increase of mosquitoes in the selected localities after the historic flooding in 2014, which will allow evaluation of the impacts of macrophyte control actions developed by the Santo Antônio hydroelectric plant after 2015. The data obtained will also help with valuable information for planning *Mansonia* control strategies.

## Materials and Methods

### Study Site

**Fig. 1.**
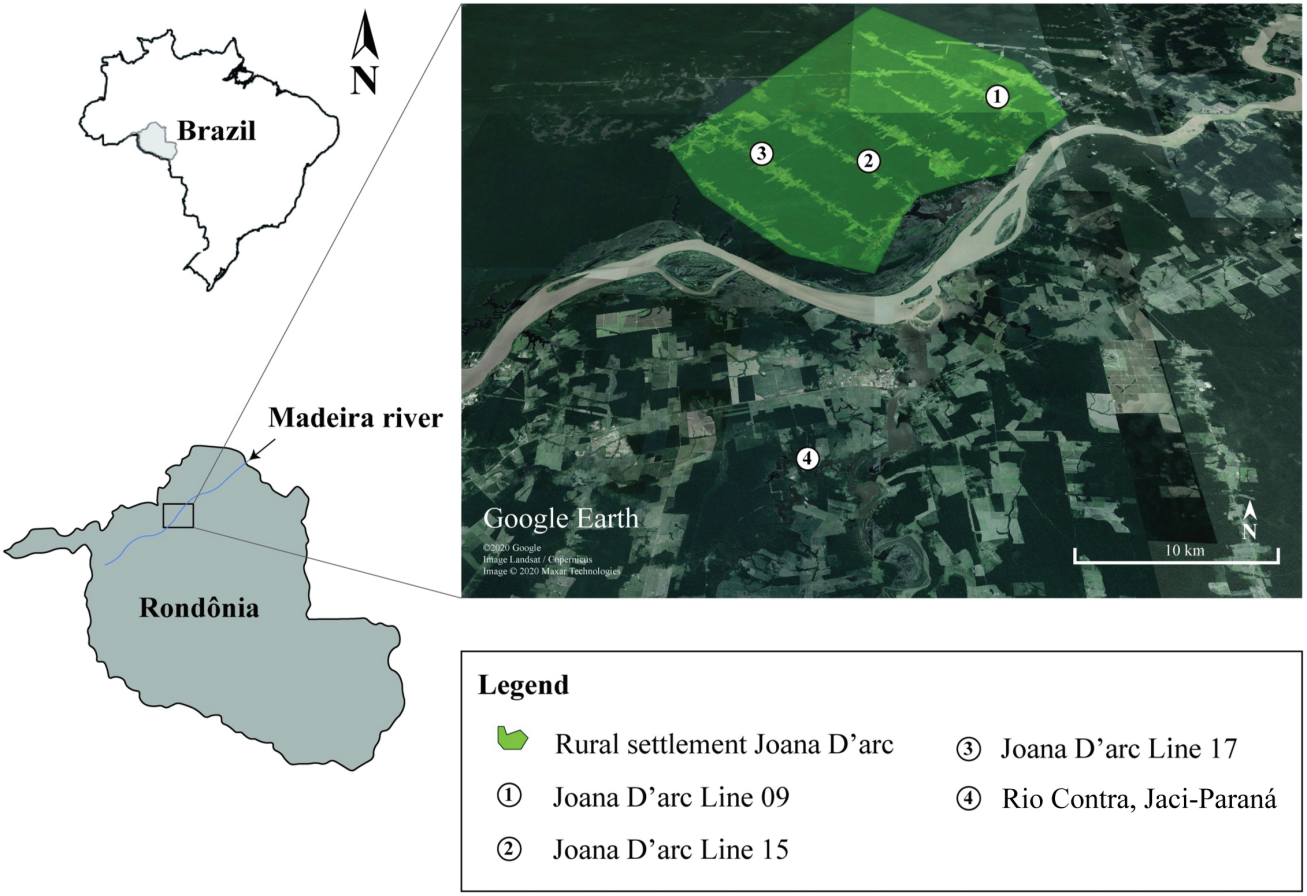
Location of sampling sites for adult *Mansonia* mosquitoes with human landing catches (HLC) in Porto Velho, State of Rondônia, Brazil.

The present study was carried out in the municipality of Porto Velho, Rondônia, Northern Brazil. The sampled locations are close to the Santo Antônio Energia (SAE) reservoir (Fig. 1). The SAE uses the hydroelectric power generation system, with low potential for altering the water flow and lower river damming, consequently, this type of dam has a lower environmental impact than conventional storage dams (Csiki and Rhoads 2010, Almeida et al. 2019). However, flooding cycles are common in the Madeira River and its tributaries. According to Köppen classification, the climate is Aw – Rainy tropical climate, with an average temperature ranging from 21°C to 34°C, with rare occasions when the temperature reaches 18°C. The photoperiod is approximately 11 hr and 4 min, with the sun rising at 7:10 a.m. and setting at 6:14 p.m. The average rainfall ranges from a maximum of 264 mm to a minimum of 17 mm per month. The rainy season is from October to April, and the dry season is from June to August, with transition periods in May and September (Ab’Sáber 2003).

Mosquitoes were captured over a period of five years, from 2015 to 2019, with three annual samplings, where the 1st sampling was done in February, 2nd sampling in June, and the 3rd sampling in October each year. The 1st sampling coincides with the rainy season or full peak of the Madeira River and its tributaries, the 2nd sampling with the dry period, and the 3rd sampling with the beginning of the rains in the region.

The collections were carried out by two collectors, simultaneously, inside and outside the homes, in four locations in the municipality of Porto Velho, Rondônia, Brazil. Three collection points were located in the rural settlement of Joana D’Arc, on the extension roads; Line 09 (08°58′38.6″S; 064°19′07.2″W). Line 15 (09°03′45.5″S; 064°25′05.1″W) and Line 17 (09°03′12.2″S; 064°29′40.0″W). The fourth site was on the opposite bank of Madeira River, in the rural area of Jaci-Paraná district, Rio Contra (09°18′35.0″S; 064°26′45.0″W). The sampled locations were spaced at 8.51 km (between line 17 and line 15), 14 km (between line 15 and line 09), and 28 km (between line 15 and Jaci-Paraná, Rio Contra), in a straight line (Fig. 1).

### Mosquito Collection and Processing

Mosquitoes were collected, simultaneously, inside and outside the homes, using the human landing catches (HLC) (Brasil 2019). The collections lasted uninterrupted for 24 hr at each sampled site to assess the biting activity of *Mansonia* spp. The sites were sampled on different and alternate days by two teams, one team each for indoor and outdoor, with a total of eight collectors, so that each collector spends a maximum of 6 hr collecting mosquitoes. After the first round of collection at the first site, the collectors changed times and places of capture (indoor switched to outdoor, and vice versa). The same pattern was maintained at the second, third, and fourth studied sites. This methodology also reduces the eventual collector effect, where one collector is more attractive to mosquitoes than other collectors. The total sampling effort was 144 hr/location/yr, or 192 hr/mo per sampling. The collection with HLC was approved by the research ethics committee of the Instituto de Pesquisas Científicas e Tecnológicas do Amapá. (CAAE No. 43415115.1.0000.0001).

The mosquitoes were collected with a glass aspirator and stored in screened cups, labeled with the hour of capture. The mosquito cups were placed in a polystyrene box for transport to the laboratory, at the SAE facilities, in Porto Velho, Rondônia. The specimens were sacrificed with ethyl acetate vapors and identified using a stereomicroscope and dichotomous keys (Lane 1953) with a taxonomic review study (Barbosa et al. 2007). Damaged mosquitoes were determined as ‘*Mansonia* sp.’. The voucher of the collected species was deposited in the scientific collection of the Institute of Scientific and Technological Research of Amapá (IEPA), as reference material for further studies in *Mansonia* taxonomy.

### Data Analysis

The human bite index (HBI), estimated by dividing the number of mosquitoes (N) in a given area, by the number of catchers (NC) and by the number of collection hours (NH) (Clements 1999), was calculated for each sampling, inside and outside the house. Subsequently, we tested the normality of our data with the Shapiro–Wilk test. Indoor and outdoor abundance were compared to test the visiting behavior hypothesis using the nonparametric Mann–Whitney test (W). For the hourly activity, we selected the two most abundant species in the study and generated radial graphs with hourly HBI values for each hour of the 24 hr of collection. Subsequently, Spearman (S) correction tests were conducted to explore any likely correlations between abundance and meteorological factors (Supp Table S1 [online only]). Then, graphs of temperature (ºC), relative air humidity (%), and accumulated monthly rainfall (mm) were compared with the HBI in each location.

All analyses, graphs and maps were developed using the R v 4.0.2 program (R Core Team 2020), and the ggplot2 package (Wickham 2011). Temperature and relative humidity data were measured with a thermohygrometer at each sampled location, and rainfall was verified at the Santo Antônio Energia meteorological station (SAE).

## Results

Overall, 96,766 specimens of the genus *Mansonia* Blanchard were collected over the five years of sampling. Six species were identified: *Ma. titillans* (Walker, 1848) [76.9% of total], *Ma. humeralis* Dyar and Knab, 1916 [13.1%], *Ma. indubitans* Dyar and Shannon, 1925 [2.8%], *Ma. pseudotitillans* (Theobald, 1901) [0.3%], *Ma. amazonensis* (Theobald, 1901) [0.2%], and *Ma. flaveola* (Coquillett, 1906) [0.02%]. 6,472 (6.7%) specimens were registered as ‘*Mansonia* sp.’ as they were damaged and made identification impossible (Table 1).

**Table 1.** Species composition and frequencies of *Mansonia* spp. collected inside and outside households, according to the capture periods. Peak of rainy: first annual sampling (February), Dry season: second annual sampling (June) and Beginning of the rainy: third annual sampling (October), from 2015 to 2019.

Species	Peak of the rainy season						Dry season						Beginning of the rainy						Total	%
	Inside	%	Outside	%	SubTotal	%	Inside	%	Outside	%	SubTotal	%	Inside	%	Outside	%	SubTotal	%		
*Mansonia titillans*	14,809	84.5	22,544	76.9	37,353	79.8	2,836	65.4	4,940	67.3	7,776	66.6	8,415	71.8	20,830	78.4	29,245	76.4	74,374	76.9
*Ma. humeralis*	1,087	6.2	2,647	9.0	3,734	7.9	269	6.2	570	7.8	839	7.2	3,014	25.7	5,109	19.2	8,123	21.2	12,696	13.1
*Ma. indubitans*	618	3.5	1,920	6.5	2,538	5.4	115	2.6	15	0.2	130	1.1	0	0.0	0	0.0	0	0.0	2,668	2.8
*Ma. pseudotitillans*	101	0.6	93	0.3	194	0.4	45	1.0	7	0.1	52	0.4	63	0.5	49	0.2	112	0.3	358	0.3
*Ma. amazonensis*	9	0.1	99	0.3	108	0.2	25	0.6	29	0.4	54	0.5	6	0.05	3	0.01	9	0.02	171	0.2
*Ma. flaveola*	0	0.0	0	0.0	0	0.0	4	0.09	14	0.2	18	0.1	0	0.0	0	0.0	0	0.0	18	0.02
*Mansonia* sp.	891	5.1	2,001	6.8	2,892	6.2	1,040	24.0	1,767	24.1	2,807	24.0	223	1.9	559	2.1	782	2.0	6,481	6.7
**Total**	**17,515**	**37.4**	**29,304**	**62.6**	**46,819**	**48.4**	**4,334**	**37.1**	**7,342**	**62.9**	**11,676**	**12.0**	**11,721**	**30.6**	**26,55**	**69.4**	**38,271**	**39.6**	**96,766**	**100**

The highest number of specimens was collected in the Joana D`Arc settlement: 81,093 (83.8%). The remaining 15,673 (16.2%) specimens were captured in Rio Contra. At the Joana D’Arc settlement, most specimens were sampled on line 17, *n* = 53,589 (55.4%), followed by line 15, *n* = 22,487 (23.2%), and line 9, *n* = 5,017 (5.2%) (Table 2). The abundance of *Mansonia* spp. along the sampling events varied with the highest number of specimens observed in the rainy season (46,819 – 48.4%), followed by the number of specimens observed in the beginning of rains, in October (38,271 – 39.6%) and, finally, in the dry season (11,676 – 12.0%) (Table 2). The first and second samples had the same richness of five species. In the beginning of rainy season, or third annual sampling, six species were recorded, including *Ma. flaveola* (Table 1). It was observed that the seasonal HBI, over the five years of study, was higher in the rainy season (HBI = 46.62, SD = 32.59), followed by the beginning of rainy season (HBI = 42.01, SD = 37.17), and lower in the dry season (HBI = 12.16, 14.83).

**Table 2.** Number, relative frequency, and frequency of *Mansonia* spp. in the three annual sampling periods. Peak of rainy: first annual sampling (February), Dry season: second annual sampling (June), and Beginning of rainy: third annual sampling (October), from 2015 to 2019.

Species	Peak of the rainy season						Dry season						Beginning of the rainy						Total	%
	Inside	%	Outside	%	SubTotal	%	Inside	%	Outside	%	SubTotal	%	Inside	%	Outside	%	SubTotal	%		
Line 17	7,047	40.2	13,303	45.4	20,350	43.5	1,255	29.0	2,083	28.4	3,338	28.6	7,459	63.6	22,442	84.5	29,901	78.1	53,589	55.4
Line 15	3,899	22.3	9,536	32.5	13,435	28.7	1,688	38.9	2,802	38.2	4,490	38.5	2,285	19.5	2,277	8.6	4,562	11.9	22,487	23.2
Rio Contra	6,309	36.0	5,987	20.4	12,296	26.3	679	15.7	962	13.1	1,641	14.1	1,104	9.4	632	2.4	1,736	4.5	15,673	16.2
Line 09	260	1.5	478	1.6	738	1.6	712	16.4	1,495	20.4	2,207	18.9	873	7.4	1,199	4.5	2,072	5.4	5,017	5.2
**Total**	**17,515**	**37.4**	**29,304**	**62.6**	**46,819**	**48.4**	**4,334**	**37.1**	**7,342**	**62.9**	**11,676**	**12.0**	**11,721**	**30.6**	**26,550**	**69.4**	**38,271**	**39.6**	**96,766**	**100**

More specimens were collected from the outdoors (65.3%) in comparison to the indoors (34.7%) (Table 2). A similar result was observed when the specimens were analyzed separately by species and season of the year, with most of the specimens occurring outdoors. The only exceptions were *Ma. pseudotitillans* and *Ma. humeralis*, which showed higher biting activity indoors on two occasions (Rio Contra and Line 15) (Table 1).

Based on the meteorological factors and the amount of *Mansonia* spp. there was a positive correlation between the relative humidity and *Ma. titillans* bites, both outdoors (*S* = 6661, *p* < 0.001), and indoors (*S* = 6774, *p* < 0.001). Another correlation was obtained between the temperature and the reduction of bites, indoors (*S* = 7795, *p* < 0.001) and outdoors (*S* = 1979, *p* < 0.001). Although some correlations were detected between the meteorological factors and, the increase or decrease of mosquito bites, all such results showed a weak correlation.

### Biting Behavior

**Fig. 2.**
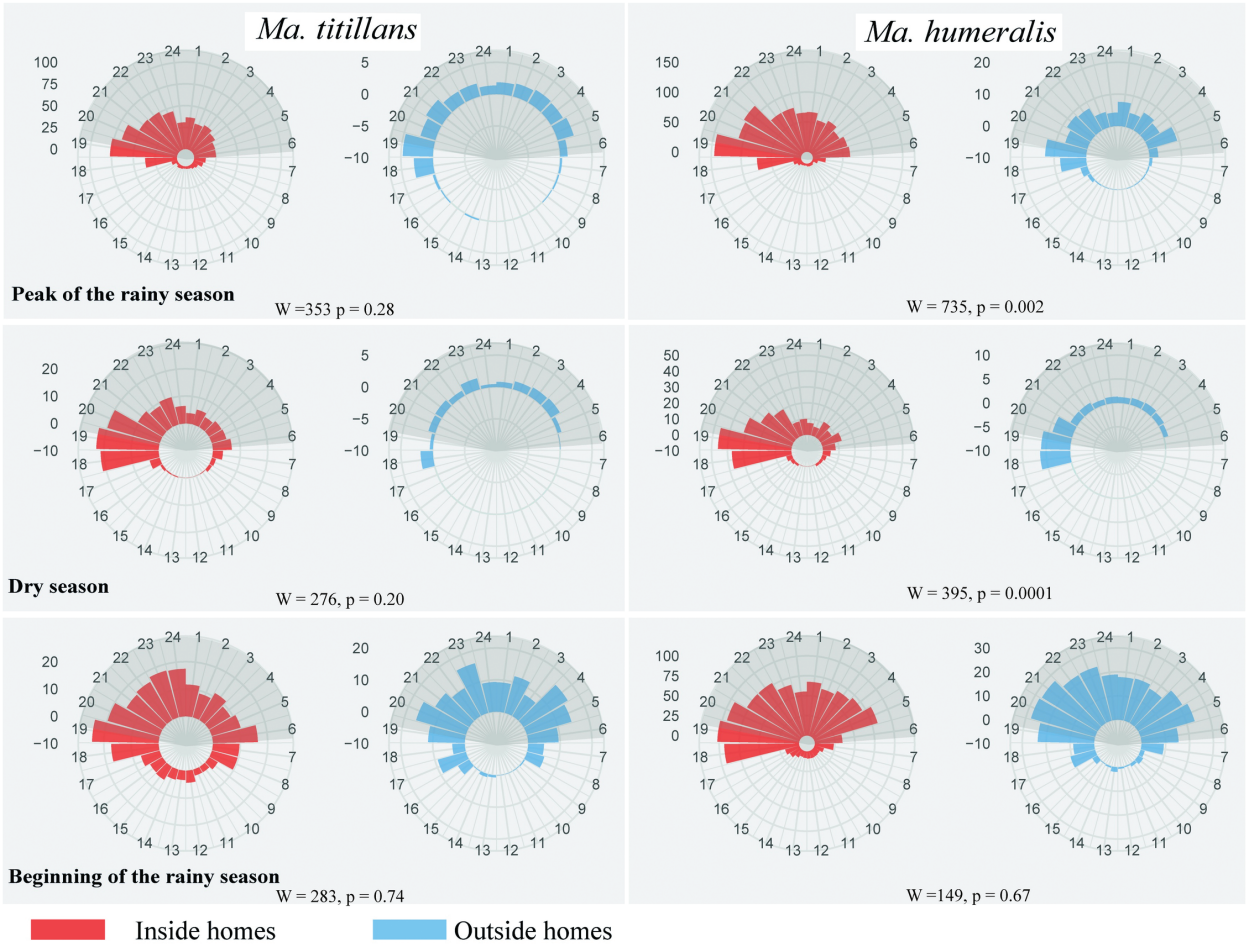
Radial bar graph (24 hr) showing activity pattern with HBI bite index for *Mansonia titillans* (left) and *Ma. humeralis* (right) for the season; rainy, dry, and early rains in the region, respectively, inside, and outside households, using human landings (HLC). The values presented refer to W = Mann–Whitney and *p*-value of significance between inside and outside by species.

The two most abundant species in the study, *Ma. titillans* and *Ma. humeralis*, were also predominantly nocturnal species (Fig. 2). Peaks in biting activity were observed in the initial hours of capture (6:00–7:00 p.m. and 7:00–8:00 p.m.) after dusk. During the rainy season (February), peak activity was observed during 7:00–8:00 p.m., with successive declines in the number of captured mosquitoes, and with higher bite indices observed outside the homes throughout the night (Supp Table S2 [online only]). The only exception regarding the predominance of mosquitoes was observed in the dry season (June) outside the homes, especially for *Ma. titillans* (Fig. 2).

In the beginning of rainy season, higher bite indices were recorded between 7:00–8:00 p.m., outside the homes (Supp Table S3 [online only]), for both *Ma. titillans* and *Ma. humeralis*. HBI were lower (HBI = 5 – *Ma. titillans* e HBI = 10 – *Ma. humeralis*) indoors, with more peaks throughout the night for *Ma. titillans*, and between 6:00–7:00 p.m. for *Ma. humeralis*. During the rainy season, the biting pattern was similar to October, with a predominance of bites outside the homes, and peak activities between 7:00–8:00 p.m. Inside the homes, *Ma. humeralis* had a higher HBI value (10) than *Ma. titillans* (HBI = 5). Throughout the day, the HBI values were low (between 5–10), with values higher in the rainy season as compared to those obtained in the dry season, especially inside the homes (Fig. 2).

### Seasonal Variation

**Fig. 3.**
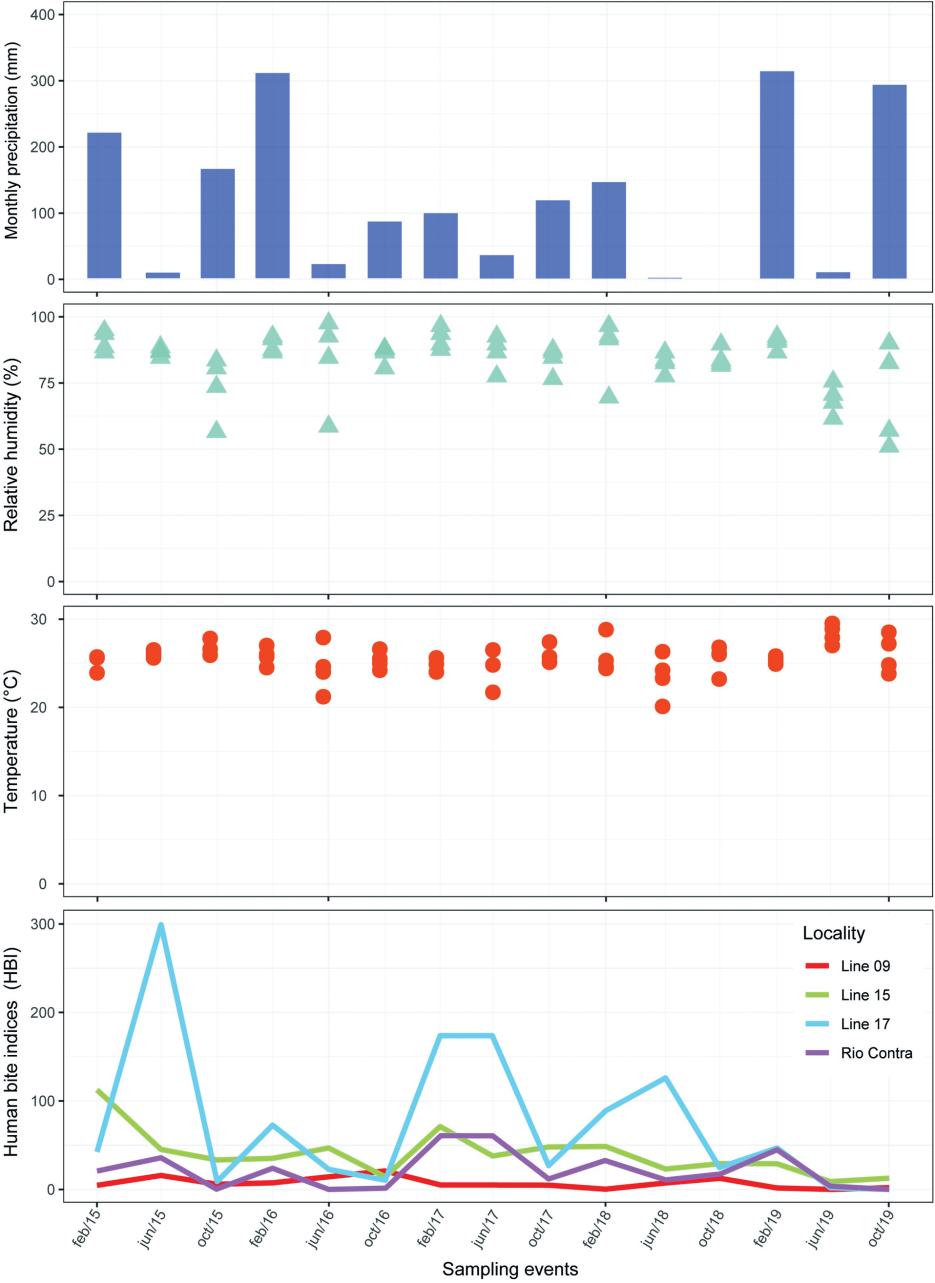
Decomposition of HBI and meteorological factors (temperature, relative humidity, and monthly accumulated rainfall) in four rural locations in Porto Velho, State of Rondônia, Brazil from 2015 to 2019.

The number of collected specimens of *Mansonia* spp. fluctuated throughout the sampling events, with the highest numbers being obtained in 2015, and the numbers of these collected specimens decreased in the consecutive years from 2016 to 2019. In addition, the highest abundance of *Mansonia* was observed in the 2nd annual sampling event (dry season) in 2015. However, from 2016 to 2019, the highest annual HBI rates were observed in the 1st annual sampling event (peak of the rainy season) each year (Fig. 3).

The decomposition of the raw data obtained for the time series and the meteorological factors are shown in Fig. 3. The adjusted dataset helped to detect a prominent peak in the 2nd annual sampling of 2015 (June/2015), and two smaller peaks in the 1st (February) and 3rd (October) 2017 samplings. A downward trend was also observed in the amount of *Mansonia* spp. in subsequent years (Fig. 3).

## Discussion

The results obtained in this study demonstrate an overview of the biting behavior of *Mansonia* in four locations near the city of Porto Velho, Rondônia. Entomological monitoring in the Amazon region generally focuses on the study of disease vectors, such as *Anopheles darlingi*, which is an important vector of malaria in the Amazon region. (Tadei 1996, Quintero et al. 1996, Ferreira 1999, Tadei and Thatcher 2000, Cruz et al. 2009). In case of genus *Mansonia*, the prerequisite of medical importance cannot be applied, as there are no occurrences in Brazil yet that can relate them to the transmission of human diseases (Atoni et al. 2019). However, due to their aggressive behavior during blood meals, this group of mosquitoes causes damage to farm animals and causes extreme nuisance for the local human population (Rueda 2007).

Entomological inventories conducted in the Amazon basin have already recorded the six species of *Mansonia* that are in the present study (Ferreira 1999, 2003; Hutchings et al. 2008, 2020). The greater diversity and abundance of the genus *Mansonia* has been reported in the areas close to white-water lakes or muddy rivers (Ferreira 1999, Araújo et al. 2020), as compared to dark water rivers, such as the Negro River (Hutchings et al. 2005, 2013, 2016). The Madeira River, characterized as a white-water river, is the largest tributary of the Amazon River basin, which is characterized by a high load of suspended sediments, alone being responsible for approximately 50% of the total suspended load transported along the Amazon River to the Atlantic Ocean (Latrubesse et al. 2005, Barbosa et al. 2007). This feature of the Madeira River may be providing adequate nutritional conditions for the proliferation of macrophytes (Chambers et al. 2007), especially on its banks and nearby lakes, formed by the natural cycle of flood and drought of the river.

*Ma. titillans* and *Ma. humeralis* were more abundant throughout the study. The prevalence of these two species is mainly due to the presence of water lettuce (*Pistia* spp.) and water hyacinth (*Eichornia crassipes* Mart, 1883) in breeding sites close to the studied area. These species of macrophyte are considered to be the preferred host plant for the larvae of *Ma. titillans* (Carpenter and LaCasse 1955). In addition, *Ma. titillans* is known to fly several kilometers in the open, flying across swamps, ponds, and lakes, to obtain blood meals or an ideal place for oviposition (Verdonschot and Besse-Lototskaya 2014). The lakes and backwaters adjacent to the Madeira River are probably the main breeding grounds for the species collected in the areas of the study. However, further investigations such as capture-mark-release-and-recapture may better assess the role of these breeding sites in maintaining local populations of *Mansonia* in the settlements.

Regarding our results of biting activity and preference for hematophagy inside or outside the homes, we observed that both *Ma. titillans* and *Ma. humeralis* are nocturnal and crepuscular, with preference for attacks outside the homes. These results did not differ from the previous studies of *Ma. titillans* from other regions of Brazil and Argentina (Consoli and Lourenço-de-Oliveira 1994, Forattini and Massad 1998, Forattini 2002, Darsie and Ward 2005, Harbach 2019), but revealed that the same behavior occurs with *Ma. humeralis*. In addition, with an uninterrupted 24-hr of sampling effort, our results present empirical evidence indicating a preference for the outdoor environment, thereby reinforcing the hypothesis of the visiting behavior (Forattini and Massad 1998), which may have direct implications for the control of adults.

The rainy season presented greater abundance of *Mansonia* spp. as compared to the dry periods and the beginning of the rains. This was also observed in the populations of *Ma. titillans* in the State of Pará (Araújo et al. 2020). In addition, our data highlights the abundance of *Mansonia* spp. collected in the early years of the study, considering that the seasonality analysis shows a downward trend in the local *Mansonia* population. Unprecedented wet conditions were reported in the summer of 2014 (December–March) in southwestern Amazonia, with a rainfall of about 100% above normal. Discharge into Madeira River (main tributary of the southern Amazon) was 74% higher than normal (58,000 m^3^/s) in Porto Velho (Espinoza et al. 2014). These flood conditions on the Madeira River may have provided viable breeding grounds for mosquitoes, especially in areas that were not previously flooded, and thus could explain the high abundance of these mosquitoes in the beginning of entomological monitoring of this study.

Other factors which may influence the occurrence of these mosquitoes in the studied areas are: deforestation, especially in recently deforested areas for pasture and logging (Ferraguti et al. 2016), hematophagic eclecticism of the species (Luz and Lourenço-de-Oliveira 1996, Kengne et al. 2003), presence of farm animals, like cattle, pigs, goats and poultry (Becker et al. 2010), high dispersal capacity of *Mansonia* spp. (Rueda 2007) and the proximity of human settlements to potential breeding sites of these mosquitoes (Forattini 2002), especially on the tributaries of the Madeira river. These local conditions can intensify the biting activity, and therefore all of the above should be considered while formulating control strategies for these mosquitoes.

In summary, the present study contributed to the knowledge of the diversity, seasonality, and biting activity of *Mansonia* in four locations in Porto Velho, in the State of Rondônia. The dominant species in the area of study, *Ma. titillans* and *Ma. humeralis*, were characterized as predominantly exophilic species, with crepuscular and nocturnal habits. The abundance of both species was higher during the rainy season in the Amazon region (October to April); however, seasonality analyses showed successive reductions in mosquito density in the subsequent years. Additional investigations would be able to elucidate the oviposition and larval rearing sites, the preferred macrophyte species, and verify dispersal routes from the breeding sites to the blood meal sources. In this way, it also would be possible to propose control actions for the immature forms and management of the main macrophyte species related to the breeding of *Mansonia* spp. in the region.

## Supplementary Data

Supplementary data are available at *Journal of Medical Entomology* online.

Supp Table 1. Weather factors collected in the field with on-site thermohydrometer, and hourly rainfall, obtained at the Santo Antônio Energia (SAE) weather station, Porto Velho, Rondônia State, Brazil.

Supp Table 2. Number of *Mansonia* spp. mosquitoes collected by hour and season, in outside the homes, using human landings (HLC), in four localities of Porto Velho, Rondônia Brazil.

Supp Table 3. Number of *Mansonia* spp. mosquitoes collected by hour and season, in inside the homes, using human landings (HLC), in four localities of Porto Velho, Rondônia Brazil.

tjac016_suppl_Supplementary_Table_S1

tjac016_suppl_Supplementary_Table_S2

tjac016_suppl_Supplementary_Table_S3
