# Supplementary material for: Seasonality and Biting Behavior of Mansonia (Diptera, Culicidae) in Rural Settlements Near Porto Velho, State of Rondônia, Brazil
Source: J Med Entomol. 2022 Feb 21;59(3):883–90. doi: 10.1093/jme/tjac016 (PMC9113164; doi:10.1093/jme/tjac016)
Supplement: tjac016_suppl_Supplementary_Table_S1 [file tjac016_suppl_supplementary_table_s1.docx]

**Supplementary table 3.** Weather data collected in the field with on-site thermohydrometer, and monthly local precipitation, obtained at the Santo Antônio Energia (SAE) Weather station, Porto Velho, Rondônia State Brazil.

| **Sampling** | **Date** | **Locality** | **Temp (ºC)** | **Relative humidity (%)** | **Local precipitation (mm)** | **Monthly precipitation (mm)** |
| --- | --- | --- | --- | --- | --- | --- |
| 1 | fev/15 | Line 09 | 23,9 | 95 | 0,04 | 219,8 |
| 1 | fev/15 | Line 15 | 25,7 | 87 | 0,03 | 219,8 |
| 1 | fev/15 | Line 17 | 25,6 | 89 | 1,0 | 219,8 |
| 1 | fev/15 | Rio Contra | 23,9 | 94 | 1,1 | 423,8 |
| 2 | jun/15 | Line 09 | 25,6 | 89 | 0,04 | 8,4 |
| 2 | jun/15 | Line 15 | 26,5 | 87 | 0 | 8,4 |
| 2 | jun/15 | Line 17 | 25,8 | 87 | 0 | 8,4 |
| 2 | jun/15 | Rio Contra | 26,2 | 85 | 0 | 36,2 |
| 3 | out/15 | Line 09 | 25,9 | 84 | 1,6 | 165,2 |
| 3 | out/15 | Line 15 | 26,6 | 81 | 0,5 | 165,2 |
| 3 | out/15 | Line 17 | 27,8 | 74 | 0 | 165,2 |
| 3 | out/15 | Rio Contra | 31,7 | 57 | 0 | 103 |
| 4 | fev/16 | Line 09 | 26,0 | 93 | 2,0 | 310 |
| 4 | fev/16 | Line 15 | 24,5 | 92 | 0,1 | 310 |
| 4 | fev/16 | Line 17 | 25,7 | 88 | 0,8 | 310 |
| 4 | fev/16 | Rio Contra | 27,0 | 87 | 0 | 341 |
| 5 | jun/16 | Line 09 | 21,2 | 85 | 0 | 21,4 |
| 5 | jun/16 | Line 15 | 24,6 | 93 | 0,9 | 21,4 |
| 5 | jun/16 | Line 17 | 24,0 | 98 | 0,1 | 21,4 |
| 5 | jun/16 | Rio Contra | 27,9 | 59 | 2,9 | 1,2 |
| 6 | out/16 | Line 09 | 24,2 | 88 | 0,01 | 85,6 |
| 6 | out/16 | Line 15 | 25,5 | 87 | 0 | 85,6 |
| 6 | out/16 | Line 17 | 26,6 | 81 | 0 | 85,6 |
| 6 | out/16 | Rio Contra | 25,0 | 88 | 0,1 | 233,8 |
| 7 | fev/17 | Line 09 | 24,0 | 97 | 0 | 98,4 |
| 7 | fev/17 | Line 15 | 24,9 | 90 | 0,6 | 98,4 |
| 7 | fev/17 | Line 17 | 25,6 | 88 | 0,02 | 98,4 |
| 7 | fev/17 | Rio Contra | 24,0 | 94 | 0,03 | 98,4 |
| 8 | jun/17 | Line 09 | 21,7 | 87 | 0 | 35,2 |
| 8 | jun/17 | Line 15 | 24,8 | 90 | 0,4 | 35,2 |
| 8 | jun/17 | Line 17 | 26,5 | 78 | 0,008 | 35,2 |
| 8 | jun/17 | Rio Contra | 24,8 | 93 | 0,007 | 35,2 |
| 9 | out/17 | Line 09 | 25,7 | 88 | 0 | 118 |
| 9 | out/17 | Line 15 | 27,4 | 77 | 0 | 118 |
| 9 | out/17 | Line 17 | 25,1 | 87 | 0,3 | 118 |
| 9 | out/17 | Rio Contra | 25,1 | 85 | 1,4 | 118 |
| 10 | fev/18 | Line 09 | 28,8 | 70 | 11 | 145,6 |
| 10 | fev/18 | Line 15 | 24,6 | 97 | 0 | 145,6 |
| 10 | fev/18 | Line 17 | 24,4 | 93 | 0,9 | 145,6 |
| 10 | fev/18 | Rio Contra | 25,3 | 92 | 0 | 145,6 |
| 11 | jun/18 | Line 09 | 23,3 | 87 | 0,05 | 1 |
| 11 | jun/18 | Line 15 | 20,1 | 83 | 0 | 1 |
| 11 | jun/18 | Line 17 | 24,2 | 78 | 0 | 1 |
| 11 | jun/18 | Rio Contra | 26,3 | 84 | 0 | 1 |
| 12 | out/18 | Line 09 | 26,0 | 84 | 0 | 0 |
| 12 | out/18 | Line 15 | 26,8 | 83 | 0 | 0 |
| 12 | out/18 | Line 17 | 26,1 | 82 | 0 | 0 |
| 12 | out/18 | Rio Contra | 23,2 | 90 | 0 | 0 |
| 13 | fev/19 | Line 09 | 25,4 | 92 | 0 | 313 |
| 13 | fev/19 | Line 15 | 25,8 | 91 | 0 | 313 |
| 13 | fev/19 | Line 17 | 25,3 | 93 | 0,5 | 313 |
| 13 | fev/19 | Rio Contra | 24,9 | 87 | 0,05 | 313 |
| 14 | jun/19 | Line 09 | 27,9 | 62 | 0,9 | 9,4 |
| 14 | jun/19 | Line 15 | 28,9 | 68 | 0,6 | 9,4 |
| 14 | jun/19 | Line 17 | 29,5 | 71 | 0,5 | 9,4 |
| 14 | jun/19 | Rio Contra | 27,0 | 76 | 0 | 9,4 |
| 15 | out/19 | Line 09 | 23,8 | 90 | 1,4 | 292,6 |
| 15 | out/19 | Line 15 | 24,8 | 83 | 0 | 292,6 |
| 15 | out/19 | Line 17 | 28,5 | 57 | 0,01 | 292,6 |
| 15 | out/19 | Rio Contra | 27,2 | 51 | 0,02 | 292,6 |
