# Supplementary material for: Seasonality and Biting Behavior of Mansonia (Diptera, Culicidae) in Rural Settlements Near Porto Velho, State of Rondônia, Brazil
Source: J Med Entomol. 2022 Feb 21;59(3):883–90. doi: 10.1093/jme/tjac016 (PMC9113164; doi:10.1093/jme/tjac016)
Supplement: tjac016_suppl_Supplementary_Table_S2 [file tjac016_suppl_supplementary_table_s2.docx]

**Supplementary Table S2.** Number of *Mansonia* spp. mosquitoes collected per hour and season, outside the homes, using human landing catches (HLC), in four localities of Porto Velho, Rondônia Brazil.

| **Hours** | **Peak of the rainy season** | | | | | **Subtotal** | **Dry season** | | | | | **Subtotal** | **Beginning of the rainy season** | | | | | **Subtotal** | **Total** | **%** |
| --- | --- | --- | --- | --- | --- | --- | --- | --- | --- | --- | --- | --- | --- | --- | --- | --- | --- | --- | --- | --- |
|  | **2015** | **2016** | **2017** | **2018** | **2019** |  | **2015** | **2016** | **2017** | **2018** | **2019** |  | **2015** | **2016** | **2017** | **2018** | **2019** |  |  |  |
| 6:00 PM | 1,145 | 540 | 514 | 144 | 150 | 2,493 | 176 | 276 | 363 | 480 | 16 | 1,311 | 464 | 195 | 666 | 432 | 373 | 2,130 | 5,934 | 9.4 |
| 7:00 PM | 1,179 | 398 | 806 | 355 | 170 | 2,908 | 63 | 362 | 542 | 426 | 14 | 1,407 | 1,012 | 347 | 888 | 944 | 420 | 3,611 | 7,926 | 12.5 |
| 8:00 PM | 1,027 | 222 | 699 | 467 | 105 | 2,520 | 76 | 173 | 398 | 254 | 12 | 913 | 488 | 365 | 773 | 722 | 191 | 2,539 | 5,972 | 9.4 |
| 9:00 PM | 847 | 168 | 573 | 460 | 120 | 2,168 | 45 | 111 | 331 | 158 | 3 | 648 | 625 | 223 | 685 | 810 | 195 | 2,538 | 5,354 | 8.5 |
| 10:00 PM | 923 | 125 | 566 | 465 | 43 | 2,122 | 43 | 120 | 242 | 118 | 4 | 527 | 264 | 175 | 602 | 522 | 326 | 1,889 | 4,538 | 7.2 |
| 11:00 PM | 775 | 122 | 460 | 396 | 103 | 1,856 | 39 | 93 | 53 | 64 | 4 | 253 | 401 | 238 | 498 | 450 | 165 | 1,752 | 3,861 | 6.1 |
| 12:00 AM | 621 | 64 | 448 | 314 | 99 | 1,546 | 17 | 74 | 113 | 86 | 7 | 297 | 265 | 205 | 481 | 356 | 263 | 1,570 | 3,413 | 5.4 |
| 1:00 AM | 866 | 93 | 416 | 282 | 72 | 1,729 | 8 | 31 | 107 | 83 | 1 | 230 | 533 | 256 | 422 | 200 | 128 | 1,539 | 3,498 | 5.5 |
| 2:00 AM | 778 | 95 | 381 | 311 | 44 | 1,609 | 6 | 22 | 83 | 96 | 5 | 212 | 422 | 166 | 388 | 216 | 116 | 1,308 | 3,129 | 5.0 |
| 3:00 AM | 867 | 109 | 424 | 280 | 27 | 1,707 | 34 | 15 | 174 | 123 | 7 | 353 | 394 | 298 | 433 | 222 | 190 | 1,537 | 3,597 | 5.7 |
| 4:00 AM | 928 | 108 | 418 | 382 | 44 | 1,880 | 36 | 34 | 81 | 117 | 4 | 272 | 193 | 268 | 429 | 222 | 220 | 1,332 | 3,484 | 5.5 |
| 5:00 AM | 1,063 | 156 | 500 | 404 | 12 | 2,135 | 97 | 52 | 102 | 63 | 5 | 319 | 283 | 427 | 517 | 128 | 163 | 1,518 | 3,972 | 6.3 |
| 6:00 AM | 558 | 103 | 140 | 288 | 2 | 1,091 | 108 | 21 | 27 | 15 | 4 | 175 | 752 | 124 | 462 | 2 | 78 | 1,418 | 2,684 | 4.2 |
| 7:00 AM | 388 | 80 | 232 | 134 | 2 | 836 | 78 | 5 | 11 | 32 | 1 | 127 | 248 | 56 | 214 | 0 | 6 | 524 | 1,487 | 2.4 |
| 8:00 AM | 217 | 10 | 20 | 42 | 0 | 289 | 54 | 0 | 0 | 13 | 0 | 67 | 71 | 13 | 170 | 0 | 0 | 254 | 610 | 1.0 |
| 9:00 AM | 185 | 0 | 35 | 14 | 0 | 234 | 33 | 0 | 0 | 9 | 0 | 42 | 33 | 0 | 44 | 0 | 0 | 77 | 353 | 0.6 |
| 10:00 AM | 188 | 0 | 10 | 3 | 0 | 201 | 28 | 0 | 0 | 4 | 0 | 32 | 55 | 0 | 41 | 0 | 0 | 96 | 329 | 0.5 |
| 11:00 AM | 184 | 0 | 6 | 6 | 0 | 196 | 0 | 0 | 0 | 0 | 0 | 0 | 54 | 0 | 47 | 0 | 0 | 101 | 297 | 0.5 |
| 12:00 PM | 190 | 0 | 5 | 3 | 0 | 198 | 0 | 0 | 0 | 0 | 0 | 0 | 65 | 4 | 23 | 0 | 0 | 92 | 290 | 0.5 |
| 1:00 PM | 191 | 0 | 4 | 9 | 0 | 204 | 0 | 0 | 0 | 0 | 0 | 0 | 38 | 9 | 16 | 0 | 0 | 63 | 267 | 0.4 |
| 2:00 PM | 144 | 0 | 9 | 9 | 0 | 162 | 0 | 0 | 0 | 0 | 2 | 2 | 36 | 18 | 21 | 0 | 0 | 75 | 239 | 0.4 |
| 3:00 PM | 190 | 0 | 24 | 4 | 1 | 219 | 23 | 0 | 0 | 3 | 0 | 26 | 33 | 4 | 62 | 0 | 0 | 99 | 344 | 0.5 |
| 4:00 PM | 249 | 35 | 25 | 39 | 0 | 348 | 37 | 4 | 0 | 8 | 0 | 49 | 108 | 25 | 59 | 0 | 0 | 192 | 589 | 0.9 |
| 5:00 PM | 296 | 50 | 48 | 208 | 0 | 602 | 49 | 0 | 21 | 2 | 8 | 80 | 199 | 19 | 89 | 0 | 40 | 347 | 1029 | 1.6 |
| **Total** | **13,999** | **2,478** | **6,763** | **5,019** | **994** | **29,253** | **1,050** | **1,393** | **2,648** | **2,154** | **97** | **7,342** | **7,036** | **3,435** | **8,030** | **5,226** | **2,874** | **26,601** | **63,196** | **100** |
