# Supplementary material for: Seasonality and Biting Behavior of Mansonia (Diptera, Culicidae) in Rural Settlements Near Porto Velho, State of Rondônia, Brazil
Source: J Med Entomol. 2022 Feb 21;59(3):883–90. doi: 10.1093/jme/tjac016 (PMC9113164; doi:10.1093/jme/tjac016)
Supplement: tjac016_suppl_Supplementary_Table_S3 [file tjac016_suppl_supplementary_table_s3.docx]

**Supplementary Table S3.** Number of *Mansonia* spp. mosquitoes collected per hour and season, inside the homes, using human landing catches (HLC), in four localities of Porto Velho, Rondônia Brazil.

| **Hours** | **Peak of the rainy season** | | | | | **Subtotal** | **Dry season** | | | | | **Subtotal** | **Beginning of the rainy season** | | | | | **Subtotal** | **Total** | **%** |
| --- | --- | --- | --- | --- | --- | --- | --- | --- | --- | --- | --- | --- | --- | --- | --- | --- | --- | --- | --- | --- |
|  | **2015** | **2016** | **2017** | **2018** | **2019** |  | **2015** | **2016** | **2017** | **2018** | **2019** |  | **2015** | **2016** | **2017** | **2018** | **2019** |  |  |  |
| 6:00 PM | 246 | 196 | 599 | 109 | 22 | 1,172 | 143 | 64 | 180 | 265 | 72 | 724 | 59 | 263 | 599 | 140 | 57 | 1,118 | 3,014 | 9.0 |
| 7:00 PM | 145 | 213 | 713 | 206 | 101 | 1,378 | 126 | 56 | 235 | 174 | 35 | 626 | 94 | 334 | 726 | 432 | 112 | 1,698 | 3,702 | 11.0 |
| 8:00 PM | 127 | 88 | 678 | 296 | 60 | 1,249 | 118 | 68 | 206 | 102 | 14 | 508 | 63 | 94 | 678 | 510 | 72 | 1,417 | 3,174 | 9.5 |
| 9:00 PM | 118 | 71 | 507 | 216 | 46 | 958 | 57 | 58 | 89 | 101 | 16 | 321 | 85 | 157 | 507 | 264 | 129 | 1,142 | 2,421 | 7.2 |
| 10:00 PM | 127 | 77 | 537 | 172 | 23 | 936 | 48 | 36 | 94 | 125 | 10 | 313 | 70 | 184 | 537 | 232 | 70 | 1,093 | 2,342 | 7.0 |
| 11:00 PM | 428 | 68 | 312 | 238 | 106 | 1,152 | 62 | 34 | 100 | 127 | 10 | 333 | 39 | 219 | 312 | 364 | 64 | 998 | 2,483 | 7.4 |
| 12:00 AM | 457 | 25 | 339 | 173 | 27 | 1,021 | 27 | 29 | 50 | 72 | 7 | 185 | 29 | 199 | 340 | 104 | 49 | 721 | 1,927 | 5.7 |
| 1:00 AM | 117 | 55 | 340 | 156 | 56 | 724 | 8 | 13 | 46 | 94 | 8 | 169 | 24 | 248 | 341 | 144 | 50 | 807 | 1,700 | 5.1 |
| 2:00 AM | 65 | 37 | 312 | 134 | 130 | 678 | 34 | 5 | 64 | 76 | 11 | 190 | 24 | 219 | 314 | 82 | 31 | 670 | 1,538 | 4.6 |
| 3:00 AM | 118 | 77 | 358 | 129 | 15 | 697 | 21 | 9 | 37 | 113 | 2 | 182 | 34 | 205 | 358 | 114 | 52 | 763 | 1,642 | 4.9 |
| 4:00 AM | 93 | 73 | 252 | 276 | 43 | 737 | 24 | 13 | 39 | 117 | 13 | 206 | 20 | 242 | 254 | 88 | 102 | 706 | 1,649 | 4.9 |
| 5:00 AM | 110 | 118 | 289 | 264 | 10 | 791 | 21 | 11 | 73 | 34 | 4 | 143 | 18 | 174 | 289 | 52 | 50 | 583 | 1,517 | 4.5 |
| 6:00 AM | 535 | 45 | 262 | 96 | 8 | 946 | 80 | 18 | 32 | 37 | 10 | 177 | 150 | 91 | 304 | 0 | 48 | 593 | 1,716 | 5.1 |
| 7:00 AM | 353 | 25 | 112 | 43 | 1 | 534 | 54 | 8 | 10 | 9 | 0 | 81 | 117 | 38 | 273 | 0 | 5 | 433 | 1,048 | 3.1 |
| 8:00 AM | 429 | 15 | 95 | 11 | 0 | 550 | 28 | 0 | 1 | 11 | 0 | 40 | 121 | 8 | 95 | 0 | 0 | 224 | 814 | 2.4 |
| 9:00 AM | 90 | 0 | 73 | 3 | 0 | 166 | 22 | 0 | 0 | 2 | 0 | 24 | 57 | 0 | 85 | 0 | 0 | 142 | 332 | 1.0 |
| 10:00 AM | 61 | 0 | 39 | 2 | 0 | 102 | 2 | 0 | 0 | 0 | 0 | 2 | 56 | 0 | 39 | 0 | 0 | 95 | 199 | 0.6 |
| 11:00 AM | 51 | 0 | 48 | 1 | 0 | 100 | 0 | 0 | 0 | 0 | 0 | 0 | 15 | 0 | 49 | 0 | 0 | 64 | 164 | 0.5 |
| 12:00 PM | 149 | 0 | 49 | 1 | 0 | 199 | 0 | 0 | 0 | 0 | 0 | 0 | 3 | 4 | 49 | 0 | 0 | 56 | 255 | 0.8 |
| 1:00 PM | 139 | 0 | 26 | 1 | 0 | 166 | 0 | 0 | 0 | 0 | 0 | 0 | 6 | 18 | 26 | 0 | 0 | 50 | 216 | 0.6 |
| 2:00 PM | 132 | 0 | 48 | 8 | 0 | 188 | 4 | 0 | 0 | 0 | 0 | 4 | 5 | 12 | 52 | 0 | 0 | 69 | 261 | 0.8 |
| 3:00 PM | 182 | 0 | 61 | 5 | 0 | 248 | 5 | 0 | 0 | 3 | 0 | 8 | 11 | 4 | 65 | 0 | 0 | 80 | 336 | 1.0 |
| 4:00 PM | 202 | 0 | 28 | 84 | 0 | 314 | 11 | 0 | 0 | 7 | 0 | 18 | 9 | 8 | 30 | 0 | 0 | 47 | 379 | 1.1 |
| 5:00 PM | 268 | 18 | 53 | 161 | 0 | 500 | 13 | 17 | 42 | 6 | 2 | 80 | 19 | 64 | 65 | 0 | 13 | 161 | 741 | 2.2 |
| **Total** | **4,742** | **1,201** | **6,130** | **2,785** | **648** | **15,506** | **908** | **439** | **1,298** | **1,475** | **214** | **4,334** | **1,128** | **2,785** | **6,387** | **2,526** | **904** | **13,730** | **33,570** | **100** |
